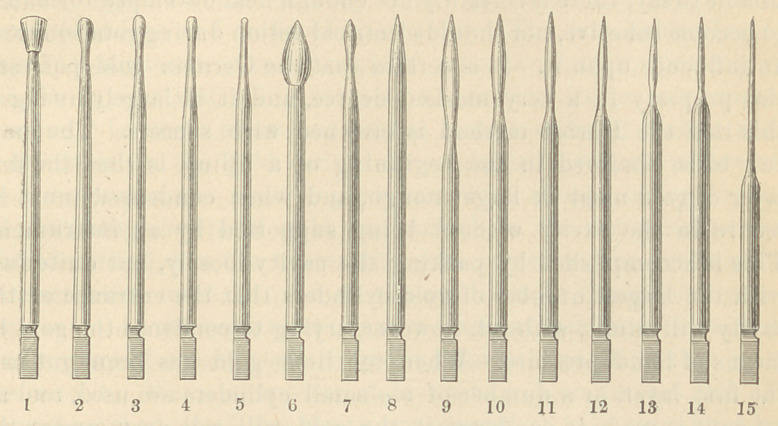# The Herbst Method of Filling Teeth

**Published:** 1884-11

**Authors:** C. F. W. Bödecker

**Affiliations:** New York


### Independent Practitioner.

VOL. V. NOVEMBER, 1884. No. 11.

### Original Communications.

### THE HERBST METHOD OF FILLING TEETH.

BY C. F. W. BÖDECKER, D. D. S., NEW YORK.

For the last two years a new method of packing gold, tin, and amalgam into cavities of teeth has been discussed in the majority of dental journals, and much has been said against the practicability of this method. During my visit to Germany I made it my special object to inquire, as far as possible, into the merits and demerits of this invention, and have, to a limited extent, employed it in my own practice. The inventor, Herr Wilhelm Herbst, an ingenious dentist of Bremen, Germany, who has practiced this method for nearly six years, is able to apply it in every cavity, and with apparent success; but whether the gold of contour fillings introduced in this manner will stand the wear as well as those made by the mallet, I am unable to say. But the method, even as it is now, in its infancy, will, I believe, when understood and applied to suitable cavities, not only save the operator a great deal of time and trouble, but will provide a better adaptation to the walls of the cavity than is possible by any other method, unless at the expense of a great deal of time, care, and skill.

While visiting the inventor I made a series of experiments, which (although I have lost the record of the exact time occupied, and their comparative weights) may be of some practical value here. I

will therefore, before discussing the method, very briefly describe some of these experiments. For the desired purpose we made use of a matrix composed of two pieces of steel joined together by a pin, which, when put together, resembled somewhat a bicuspid tooth with a large, deep cavity in its grinding surface. Into this cavity several rather deep pits were drilled all around its wall. No dental practitioner would make such excavations in the wall of a tooth, but for the sake of testing what the new method would accomplish we proceeded in that way. We alternately filled this cavity in different ways, and with different preparations of gold, Herbst employing his method, while I made use of the Bonwill mechanical mallet. The experiments were made with gold foil in the form of cylinders, folded foil, and foil twisted into the form of a rope and cut into pellets. The first gold used was that of Carl Wolrab, of Bremen, Germany, and the cylinders were used without being heated. The time required by Herbst to fill the steel cavity was about twelve minutes, and when the matrix was separated the plug was found to be perfect, even in the deepest pits. Although the gold had not been annealed, and showed no signs of cohesion before packing, not a particle could, by ordinary pressure with the fingers, be broken off. This proved that the several cylinders were united to a comparatively solid mass, which upon hammering proved to be quite malleable. The introduction of the same gold into the same cavity by the Bonwill mechanical mallet occupied, I believe, about thirty minutes, but when the matrix was separated the surface of the plug was found to be imperfect in some of the deep pits, although its weight was considerably more than that made by Herbst. Slight pressure with the fingers separated the plug into two pieces, which by hammering crumbled into several smaller ones. The other two experiments, in which the gold had been used without being brought in contact with the flame of an alcohol lamp, gave about the same results; but how different were those obtained from gold which, previous to its introduction, had been annealed In this instance the packing of the gold by rotation (the Herbst method) required more time, and yet the plug, when removed from the matrix, showed imperfections upon its surface, and its weight was less than that which had been made by unannealed gold. Although the plug could not be broken by the fingers, it separated into several pieces under the hammer. The experiments made with

Wolrab's gold when annealed and introduced by the Bonwill mallet, when compared with those of the same instrument when the unannealed gold was used, showed very little difference either in weight or in external appearance. But the several cylinders were firmly united, and the mass could be hammered without crumbling in pieces. The experiments made with the gold of our American manufacturers gave about the same results when packed with the Bonwill mallet, but very different ones when introduced by the Herbst method. In no instance was the surface of the plugs introduced by the latter as perfect as when made with Wolrab's gold, and their weight was less than that of those made by the same methods where German gold was employed, although more time was required to introduce the American preparations.

The conclusions we arrived at from these experiments were that one of the principal requirements for the successful practice of the Herbst method is a very soft quality of gold, which by rubbing with the burnishers becomes cohesive. The first layers of gold that are introduced against the walls and edges of the cavity ought not be annealed, nor even warmed, but as the filling progresses nearer to the surface, or in contour operations, slight warming or even annealing will be found necessary. The form of gold best adapted, especially for the beginning of an operation, is cylinders, and of all the preparations used there were none which worked so well as those of C. Wolrab, of Bremen, which seem to be especially adapted to this purpose. The walls and edges of a cavity can be very perfectly filled by this method, with much less trouble and care than is necessary when the mallet is employed, besides requiring less time for the introduction of the gold. Although the body of a filling made by the Herbst system is probably not quite as solid as when done by the mallet, yet the adaptation to the walls of the cavity is much better.

Before describing the method of filling different cavities I will allude to some general rules which hold good for all operations. It is almost a universal law that the filling of cavities which by the old system gave us the most trouble, are easiest to fill by the Herbst system. The uppermost layers of cavities in the grinding surfaces, however, which by the mallet system is very simple, are a little more difficult to complete by the new process. A great deal of attention must be given to the beveling of the edges in cavities that are prepared to be filled by the mallet. In the Herbst method this is not only unnecessary but objectionable, as the edges when much rounded are rather difficult to fill perfectly, unless the matrix is so adjusted that the gold extends a little out of the cavity. But the best results will be obtained, at least in the proximate surfaces of molars and bicuspids, when the edges of the cavity are made perfectly smooth and rounded off but very slightly. Nor is it necessary to make deep retaining or starting points, but the cavity should be so prepared that its general form will securely anchor the filling. When a proximate cavity has been prepared in the manner above described, and the matrix applied properly, the filling in this situation will require but very little finishing, which, especially when under the gum, is a very painful and troublesome procedure. A point of great importance in proximate cavities or contour operations is the adjustment of the matrix, which must be applied firmly enough so that it does not move during the introduction of the filling materials, for to a certain degree success is dependent upon the stability of the matrix. The object of this instrument is to convert a cavity which possesses but one, two, or three side walls into one with four side walls, and, as has been mentioned before, there will be but little finishing required after the introduction of the filling material. The matrix that is used for the most of the proximate surfaces is made from a piece of annealed watch or clock spring, or wood, but for operations where lost portions of teeth have to be restored, shellac may be employed. Although Herbst can accomplish almost everything with the matrices above mentioned, I am convinced that a screw matrix, which could be so firmly applied that motion during the introduction of the filling material would be impossible, will simplify this method in many instances. I have therefore constructed a universal screw matrix which, if it proves a success, I shall describe in a future number of this journal.

The instruments for the introduction of the filling material are mostly ordinary smooth burnishers made of steel, although I believe that any other hard material, such as blood stone, agate, etc., would be far superior to steel instruments. One of the principal objections to steel instruments is that in use they rapidly become coated with a film of gold, in which condition they cannot be employed to condense a newly added layer, as the gold on the instrument will cohere to that just added to the filling, and will consequently be pulled out of the cavity. When a layer of gold has been thoroughly condensed with a clean, bright instrument, however, it is an advantage to burnish the surface with one which is coated with gold, as an instrument when used under these circumstances will roughen the surface and make the gold cohesive. To neutralize the coating of the instrument with gold, Herbst recommends that they be rubbed upon a piece of pure block tin. But it is evident that by so doing some of the tin will adhere to the instrument and it will be incorporated in the gold of the filling, which after a time may alter the color of the finished work. I have for this purpose used, with good results, fine crocus cloth, but it requires a little more time than the use of tin. During an operation at the November or December clinic of the First District Dental Society, Dr. Wheeler, of Albany, handed me a small blood stone, which I used for burnishing on the last layers of gold. This worked better than any steel instrument I have ever used in introducing gold by rotation, as not a particle of the material adhered to the instrument.

The introduction of the gold, which is the main new feature of the Herbst method, is sometimes attended with some difficulty, but if certain general rules are observed, it becomes quite simple. It is probably owing to this that many gentlemen who have tried this method of filling teeth and failed, have done so on account of not being acquainted with the general principles of the system. During the introduction of the filling material we observe a peculiar phenomena; namely, the gold which, when unannealed, apparently shows no signs of cohesion, working as soft as tin foil, when burnished becomes quite cohesive. What can be the reason for this I am unable to say; there is certainly not enough heat developed to cause it to become cohesive, nor should electrical action during rotation exert an influence upon it. It is certain that the German gold possesses this property in a very marked degree, and it is largely owing to this that the Herbst method is crowned with success. The main rule to be observed in the beginning of a filling is that the first layer of gold must be large enough, and when condensed must lie secure in the cavity without being supported by an instrument. This is accomplished by packing the cavity loosely, but quite full, with the largest number of gold cylinders that the entrance of the cavity will admit, without, however, trying to condense the gold by means of hand pressure. When too little gold has been put into the first layer, or a number of too small cylinders are used, and an attempt is made to condense it, the gold will roll away under the instrument and become too hard to be again adapted to the walls and edges of the cavity. The same condition will be observed when the first instrument used in condensing the gold has been too small. For this purpose one of the pear-shaped instruments (Nos. 1 to 4), as large as the entrance of the cavity will permit, must be used. The first layer of gold must not be permitted to move at all during condensation by the rotatory instruments, as a failure is inevitable when the first gold introduced against the walls or edges of the cavity has been allowed to move. In using these instruments care should be taken not to run the engine too fast, nor to allow the burnisher while in motion to be in contact with the gold longer than from one to three seconds, otherwise it will heat the gold to such an extent as to cause discomfort, or even great pain to the patient. The best manner to work them is, perhaps, somewhat analogous to the action of the slow automatic mallet, where by pushing against the plugger point we obtain the blow, but after this we have to lift the instrument again to allow the spring to force the point forward. So it is with this method; first, the burnisher is pressed firmly upon the gold for a few seconds, after which it must be allowed to cool for a second or two. When manipulated in this manner it will be found that the heat developed is so small that it will cause no inconvenience whatever to the patient. When the gold has been burnished down into the bottom of the cavity by one of the pear-shaped instruments, it is thoroughly condensed with a roof-shaped instrument, No. 5, in such a manner that the point of a hand plugger cannot condense it any more when firmly pressed along the edges and walls of the cavity. The last named (roof-shaped) instrument is easily made of a broken bur, by putting it into the engine, and while in motion grinding its point upon an oil stone. In some situations, as the buccal walls of molars and bicuspids, when the gold cannot be condensed by direct action of the instruments, the right angle attachment should be employed. All succeeding layers of gold are packed in the same manner as above described, but the nearer the gold comes to the surface the more attention must be paid to the condition of the revolving instrument. At the beginning of every fresh layer it must be rubbed upon a piece of crocus cloth to free it from gold particles that may adhere to it. When a number of layers have been secured and all the walls and edges of the cavity are covered, it will sometimes be found necessary, if the operation is to be concluded by the Herbst system, to slightly warm or anneal the gold and press it into the required position by hand pressure before rotation upon it is commenced. With reference to the results of the experiments which I made with the inventor, I deem it safer, especially for a beginner with this system, to finish an operation in the old accustomed manner. The experiments demonstrated that the walls of a cavity, when filled by the Herbst system, are more perfect, although it did not weigh quite as much as the one made by the mallet, which showed imperfections upon its surface. Therefore, the center of the plug made by rotation could not have been as solid as when introduced by a mallet. I admit that the specific weight of a gold plug is not of great importance, but the more solid a filling is upon its grinding surface the better it will wear. Furthermore, the adjustment of the last layers of gold requires almost as much time as when made by the mallet. As I desire that every dental practitioner should be benefited by this mode of practice, I would advise him to make some experiments upon natural teeth, set with their roots in plaster, before attempting to apply the method in the mouth of patients, although it will be found that it is easier, especially with this method, to fill a cavity in the mouth than in an experimental way out of the mouth.

Tin and gold combined, tin foil, and also amalgam, have been packed by the Herbst method with great advantage, and the inventor even claims the amalgam can be packed under saliva without impairing its durability. Whether this is so or not I am unable to state. I certainly should prefer, if possible, to have every cavity dry and well disinfected before the introduction of the filling material.

I will now describe the method for special cavities, and will begin with those which are the easiest to manipulate.

I. Distal surfaces of bicuspids and molar teeth: After the cavity has been prepared and the rubber dam adjusted, a matrix is applied, which, if the cavity to be filled faces on another tooth, is best prepared from a small piece of a previously annealed clock spring. This is placed between the two teeth, and if the separation between them is very small it may be secured by pushing two pins (one from the buccal, the other from the lingual surface near the gum) between the matrix and the adjoining tooth. In some instances the next tooth also contains a cavity of such dimensions that it is impracticable to secure the matrix firm enough by means of pins. This difficulty may be obviated by filling the opposite cavity tightly, either with cotton or previously warmed shellac (the matrix having previously been put in position). When the adjoining tooth is missing, or the space between the teeth is very large, a block of wood firmly fitted against the steel matrix may be used. If this cannot be accomplished, the loop matrix intended for use in teeth standing alone will answer the purpose. The matrix being in place and the cavity having been thoroughly disinfected and dried, it is ready for the introduction of the gold. Two, three, or four large gold cylinders are, without attempt at condensation, loosely placed in the cavity by a hand instrument. One of the pear-shaped instruments (Nos. 1 to 4), as large as the entrance of the cavity will admit, is placed in the engine, cleaned upon crocus cloth, and with it, while in rotation, the gold is compressed first into the bottom and then against the side walls of the cavity. This being done, one of the roof-shaped instruments (No. 5) is placed in the engine, and while in rotation cleaned upon an Arkansas stone. The gold is then thoroughly condensed into every depression, and especially against the matrix and edge of the cavity. In this manner layer after layer is introduced until the cavity is filled; or, as mentioned before, the last layer may be packed by the mallet.

The introduction of the gold into cavities situated in the mesial surfaces of molars and bicuspids is a little more troublesome. The anterior edges and walls of the matrix in these localities cannot always be reached by direct action ; right angle attachment in these cavities is therefore indispensable. In bicuspids and first molars the gold may be first condensed with a straight instrument, but in every layer this should be followed by a No. 5 burnisher in the right angle attachment, which while rotating is firmly pressed forward against the matrix and edge of the cavity.

The packing of gold in cavities upon the grinding surface of molars and bicuspids is somewhat different. The first layer introduced must extend over the whole surface, and be sufficiently thick to lie quietly when the instrument (No. 5) is used to condense it into the several depressions of the cavity. When the first layer has been securely condensed by this method the next one is much easier. To facilitate the packing of the succeeding layers the gold cylinders may be warmed, or even slightly annealed, and by means of hand pressure partially condensed before rotation upon them is commenced. The instrument used after the addition of every new layer must be as large as possible and absolutely clean, and be applied (especially at first) with considerable pressure. When the operation is nearly completed I would advise, especially for beginners, that the operation be finished by the mallet.

Proximate surfaces of the incisors can be very easily and quickly filled by this method. The cavities are prepared in the same manner as for filling by any other system, with the exception that no starting points are made; a slight round undercut at the cervical wall and one toward the cutting edge of the cavity is amply sufficient. The separation required for this method is not more than when the cavity has been prepared for other methods. Herbst fills all these cavities with a No. 5 instrument, but I have lately used a burnisher made of a small round bur, which worked very satisfactorily. The introduction of the first layer of gold is materially the same as in former instances, but the uppermost layers are a little differently manipulated, especially when there are two cavities to be filled which face each other. Such cavities (as Herbst suggests) may be filled in the following manner: When the first layer in both cavities has been thoroughly condensed, more gold cylinders are added in both cavities and condensed as though these were but one.

When sufficient gold has been introduced the two fillings are separated by No. 14 (an ordinary fine sewing needle secured in a mandrel or needle chuck), which while rotating is pressed through the median line of the fillings in several places. The two fillings are further separated by means of a thin clock-spring saw, the edges thoroughly condensed with one of the pointed burnishers (Nos. 12 to 14), and finished in the accustomed manner.

Proximate surfaces of the incisors, when their lingual walls are broken, are comparatively easy to manipulate. In these instances a matrix is applied to the lingual wall, which may be made in the following manner: A piece of shellac the size of a large walnut is warmed over an alcohol lamp to the consistency of putty, and pressed against the lingual wall, extending a little over the cutting edge of the four or six front teeth. If any of the shellac is pressed into the cavity it must be carefully removed by cold excavators while in position, or the matrix after it has become hard may be removed from the mouth, trimmed as desired, and put back again into its place. When the labial wall of such a cavity is broken to such an extent that the gold can be easily packed from the labial surface, an additional steel matrix must be applied against the proximate surface of the cavity. This matrix may be secured by either pins, wood, cotton, or it may be warmed and pressed into the shellac matrix of the lingual surface. This steel matrix (made of a piece of clock spring) must not quite reach to the labial surface of the tooth to be filled, as it may offer an obstruction to the introduction of the rotating instrument. No difficulty will be found in the introduction of the gold, as its manipulation is carried on in the usual manner.

Contour operations of the cutting edges of the front teeth have only been accomplished by Herbst within a comparatively short time. Little, therefore, can be said of their practicability, although they require comparatively little time. One of the preparations sent to me by the inventor, involving the mesial, distal, and about one-sixteenth of an inch of the cutting edge of a lower incisor, only required forty minutes' time for the introduction of the gold. The principal difficulty in these operations is the making of a proper matrix. When this has been accomplished the filling is comparatively a simple matter. A matrix is prepared of warmed shellac, which (as described above) in this condition is pressed behind the lingual walls of the four or six front teeth, extending for a little distance over their cutting edges. The proximate walls are enclosed by pieces of clock spring, adjusted on both sides and, when possible, fastened independently as well as into the shellac matrix of the lingual wall. A third piece of clock spring is then adjusted across the cutting edge of the tooth to be restored. When thus arranged these matrices form a simple cavity, with four side walls, into which the gold is easily packed.

## Figures and Tables

**Figure f1:**